# Nitrous Oxide Gas—the Phonograph as a Reporter

**Published:** 1890-01

**Authors:** S. W. Sanford

**Affiliations:** Henning, Tenn.


In the *Reporter* of November 2d, you mention a death from nitrous oxide gas, or rather from apoplexy following administration of the gas. Several years ago I administered nitrous oxide gas to a lady, for a dentist to extract some teeth, and she came near dying from what I then considered threatened apoplexy. The case you mentioned reminded me very forcibly of my experience then, and I hope never to have a repetition of it. In the same issue of the *Reporter*, Dr. A. Hamilton Deekens reports the union of a cut-off finger, which reminds me of what happened to an old physician many years ago; long before antiseptic surgery was practiced. A man with a finger cut off came to him bringing the finger. The doctor was drunk and sewed the finger back. It united nicely. But lo! the doctor had sewed it on with the palm surface turned the wrong way. The doctor, after sobering up, wanted to amputate the finger and try to put it back right, but the patient declined and the doctor was annoyed many years by having his mistake constantly exhibited as a great curiosity, Yours truly,

S. W. Sanford, M.D.

Henning, Tenn., Nov. 5, 1889.

---

The latest use to which the phonograph has been put is the recording of discussions. Quite recently a paper to be presented at the First District Dental Society of New York State, was privately read and discussed before some Philadelphia dentists in this city, over a phonograph. The cylinders were sent to New York and the discussion repeated after the reading of the paper.

